# Study on hydration cementation and enhancement mechanism of grout-rock interface of PVA modified grouting material

**DOI:** 10.1371/journal.pone.0362466

**Published:** 2026-11-05

**Authors:** Youwei Li, Haoyu Hu, Yujun Zhang, Gui Liu

**Affiliations:** 1 CCTEG Coal Mining Research Institute, Beijing, China; 2 Mining Research Branch, China coal Research Institute, Beijing, China; King Mongkut's University of Technology North Bangkok, THAILAND

## Abstract

The weakly cemented sandstone aquifer in coal mine is prone to particle softening under mining disturbance and water-rock interaction, which induces the decrease of rock integrity and the development of water conducting channel, leading to the instability and failure of aquifer. To address the low early-age hydration activity of grouts containing a high proportion of coal gangue, discontinuous grout-rock interfacial bonding, and limited adaptability to weakly cemented sandstone, this paper uses cement and coal gangue(CG) as raw materials to prepare modified grouting materials with different polyvinyl alcohol (PVA) content. The uniaxial compression, XRD, FTIR and SEM test methods were used to systematically study the influence of PVA content on the performance of solid waste based slurry, the bearing characteristics of slurry-rock composite, the evolution of hydration products and the interface micro structure. The synergistic enhancement mechanism of PVA induced hydration cementation film coating and slurry-rock interface was revealed. The results show that: (1) High content of CG weakens the early hydration activity of cement slurry system. After PVA is added, the fluidity of slurry increases, the initial and final setting time is further prolonged, and the bleeding rate decreases, indicating that PVA can improve the flow stability and water retention performance of coal-based solid waste slurry. (2) PVA content has obvious dosage dependence on the bearing capacity of the grout rock complex. The peak stresses of the 0%, 0.3%, 0.6% and 1.0% PVA groups are 4.87 ± 0.14 MPa, 5.35 ± 0.06 MPa, 6.04 ± 0.09 MPa and 5.12 ± 0.09 MPa, respectively. The 0.6% PVA group shows relatively better mechanical properties within the test range of this paper. (3) Appropriate amount of PVA can promote the filling of C-S-H gel, AFt rod crystals and C-H flake crystals on the surface of sandstone particles and in the pores and fracture, and adsorb and coordinate through hydroxyl groups to form hydration products and flexible polymer film. The hydration products provide a rigid skeleton, and the PVA flexible film plays a buffering role in the interface bonding and deformation, and synergistically realizes the densification, continuity and toughness enhancement of the weakly cemented sandstone-grout interface. The experimental results show that 0.6% PVA content can achieve a better balance between slurry performance, water retention stability, interface hydration cementation and mechanical bearing capacity, which can provide material design basis for grouting reinforcement of weakly cemented aquifer, thickening of aquiclude and prevention and control of mining-induced water disasters.

## Introduction

Under the influence of China ‘s regional economic development, the focus of coal mining has begun to shift, and the construction of five major coal supply security bases in Shanxi, western Inner Mongolia, eastern Inner Mongolia, northern Shaanxi and Xinjiang has continued to advance. The main mining areas in western Inner Mongolia and Shaanxi have been proved to have abundant coal reserves and low overall mining degree, but the coal-forming mode, sedimentary environment and tectonic background have changed [1–3]. Some mines have weakly cemented sandstone strata, and the rock mass is generally soft and the degree of cementation is poor [4]. The difference of overburden rock characteristics and structure caused by different diagenetic environments is an important challenge for coal mining in western China [5,6]. Rock mass grouting technology is one of the most commonly used and effective engineering methods to control bad geological problems such as reinforcement, water plugging and seepage prevention, and rapid repair in geological engineering [7].

The development and optimization of grouting materials is the key to improve the effect of grouting reinforcement. Modern new grouting materials show a diversified development trend for complex engineering requirements [8]. For weakly cemented sandstone with high porosity, low cementation degree and significant water sensitivity, only analyzing the macroscopic flow and consolidation strength of slurry is not enough to clarify its reinforcement mechanism. It is necessary to further reveal the evolution of hydration products and the cementation behavior of slurry-rock interface after slurry enters into pore cracks [9,10]. From the perspective of the evolution from Industry 4.0 to Industry 5.0, Ayyappan et al. pointed out that the development of sustainable composite materials needs to comprehensively consider material properties, resource utilization efficiency, environmental impact and manufacturing process optimization, and promote the transformation of material system from traditional performance orientation to full life cycle sustainable design [11]. Singh et al further pointed out that artificial intelligence and digital manufacturing technology are driving the development of material composition design, performance prediction and manufacturing process control to data-driven and intelligent optimization [12]. Nanofiber materials play an effective role in stimulating the physical and chemical activity of Portland cement. Wang et al. found that PVA molecular chain contains a large number of hydroxyl groups (-OH), which can form new ions with water molecules, cement hydration products and active groups on the surface of coal gangue, enhance its adhesion and matrix stability, thus improving the dispersion and water retention of the slurry system [13]. Guo et al. [14] studied the macroscopic and microscopic strength characteristics of graphene oxide and polyvinyl alcohol fiber mixed in cement-based grouting materials. It was found that the proper amount of PVA added to the grouting material made its internal structure stable and effectively hindered the generation of cracks.Wen et al. [15] studied the effects of PVA concentration, water-cement ratio and other factors on the coagulation time, fluidity, water bleeding rate, viscosity and 28-day uniaxial compressive strength of the slurry. LI et al. [16] developed a fiber-modified coal-based solid waste grouting material with glass fiber, polyvinyl alcohol fiber and basalt fiber as reinforcing materials, which enhanced the fluidity and impermeability of the slurry. Li et al. [17] studied the mechanism of nano-SiO_2_ on the mechanical behavior of grouting bonding interface and the influence of interface micro-mechanical properties on the macroscopic mechanical properties of grouting coal. Ma et al. [18] designed a composite waterproof adhesive layer of cement-mudstone interface. The enhancement behavior was studied by uniaxial compressive strength (UCS) test, interface direct shear test, contact angle measurement and microstructure characterization.

Existing studies have confirmed that PVA can improve the mechanical properties and impermeability of cement-based materials by adjusting the hydration process of cement-based materials, improving slurry stability, and forming a flexible polymer film. However, these studies mainly focus on the macroscopic properties and hydration behavior of pastes such as ordinary cement, fly ash or ultrafine cement. There is still a lack of systematic understanding of the polymer-cement composite system under the condition of high proportion of coal gangue substitution, and the cost is still an important factor restricting the popularization and application of high molecular polymers such as PVA in grouting materials. How to optimize the PVA content and slurry ratio on the basis of both economy and feasibility, so as to give full play to the effect of interface strengthening, water retention toughening and impermeability modification, is still an urgent problem to be solved [8]. The improvement of the performance of grouting materials has shifted the focus of research and development to environmental protection and economy [19]. The technology for preparing grouting materials from coal-based solid wastes has promoted the balance between efficient utilization of solid waste and engineering performance. Zhou et al. [20,21] prepared coal gangue geopolymer grouting material with coal gangue, fly ash and cement as main materials. Through SEM electron microscope, it is found that the strength of grouting material mainly comes from C-S-H, AFT and C-(N)-S-A-H generated by CG powder, fly ash and cement. Li et al. [22] inhibited the development of cracks and the transformation of macro and micro cracks in the later stage of consolidation by adding fibers to coal-based solid waste slurry, and significantly improved the crack resistance of consolidation. Wang et al. [23] found that when the ratio of coal gangue, cement and clay is 60%:30%:10%, the strength and permeability coefficient of grouting composite can be significantly improved. Li et al. [24] took coal-based solid waste grouting material as the research object, introduced in-situ cross-linked polymer network structure, studied its regulation effect on slurry performance, macroscopic mechanical behavior, hydration product type, microscopic pore structure and carbon sequestration effect, and revealed the coupling enhancement mechanism between polymer network and solid waste-based cementitious system. Yang et al. [25] prepared a compound cement coal-based solid waste grouting material with coal gangue and fly ash as raw materials, and studied the hydration products of the grouting material under the combination of the two cements.

In summary, important progress has been made in improving the performance of grouting materials, improving the strength and impermeability of grouting materials through nanofibers, polymers and coal-based solid waste. However, the current research mostly focuses on the grouting reinforcement of hard rock fissures, and there are few studies on the grouting of weakly cemented soft rock or even loose non-diagenetic rock strata. In the grouting process of weakly cemented sandstone, the action object of PVA is no longer limited to the interior of the slurry, but also involves the migration of slurry in sandstone pores and fissures, the deposition of hydration products on the rock surface, and the regulation of polymer film on the stress transfer of slurry-rock interface. Therefore, the coupling relationship between PVA and the overall hydration of the slurry, the local slurry-rock interface structure and the macroscopic mechanical response of the composite is not clear. Therefore, this paper takes the weakly cemented sandstone aquifer of Tarangaole Coal Mine as the engineering background, takes the cement-CG solid waste-based slurry as the basic system, introduces polyvinyl alcohol as the interface control component, and sets different PVA content. The slurry fluidity, setting time, bleeding rate, uniaxial compression, XRD, FTIR and SEM tests were comprehensively carried out to construct an analytical framework for the overall phase transformation and interface structure reconstruction under PVA content. The regulation effect of PVA on the evolution of hydration products and the interfacial cementation of weakly cemented sandstone in high coal gangue system was mainly revealed, and the mechanism that the overall hydration enhancement and local interface bearing capacity were not simply synchronized was clarified. It provides important technical support for aquifer protection, safe coal mining and coordinated development of resources in coal-uranium superimposed areas.

## Test materials and methods

### Engineering background

Tarangaole Coal Mine is located in Hangjin Banner, northwest of Ordos Basin, Inner Mongolia. Sandstone, are interactively developed in the overlying strata of coal-bearing strata (Fig 1). According to the core exposure, the integrity of sandstone is poor, locally broken in blocks, broken blocks or granular, and the distribution of cement is uneven. After the cement is destroyed, the strength and water physical properties of the rock mass are significantly reduced. Weakly cemented strata are different from hard rock and loose sand layers. They have experienced late diagenesis. The porosity between strata particles is higher, the cementation is weaker, and the strength is lower. Compared with similar strata in the eastern region, the roof rock is broken more seriously during the mining process of weakly cemented overburden. It is easy to disintegrate into mud when encountering water, and the rheological properties are more significant [26–28]. Tarangaole coal mine is a coal-uranium superimposed mining area, and the average vertical distance between uranium ore body and coal seam is about 139 m.

**Fig 1:**
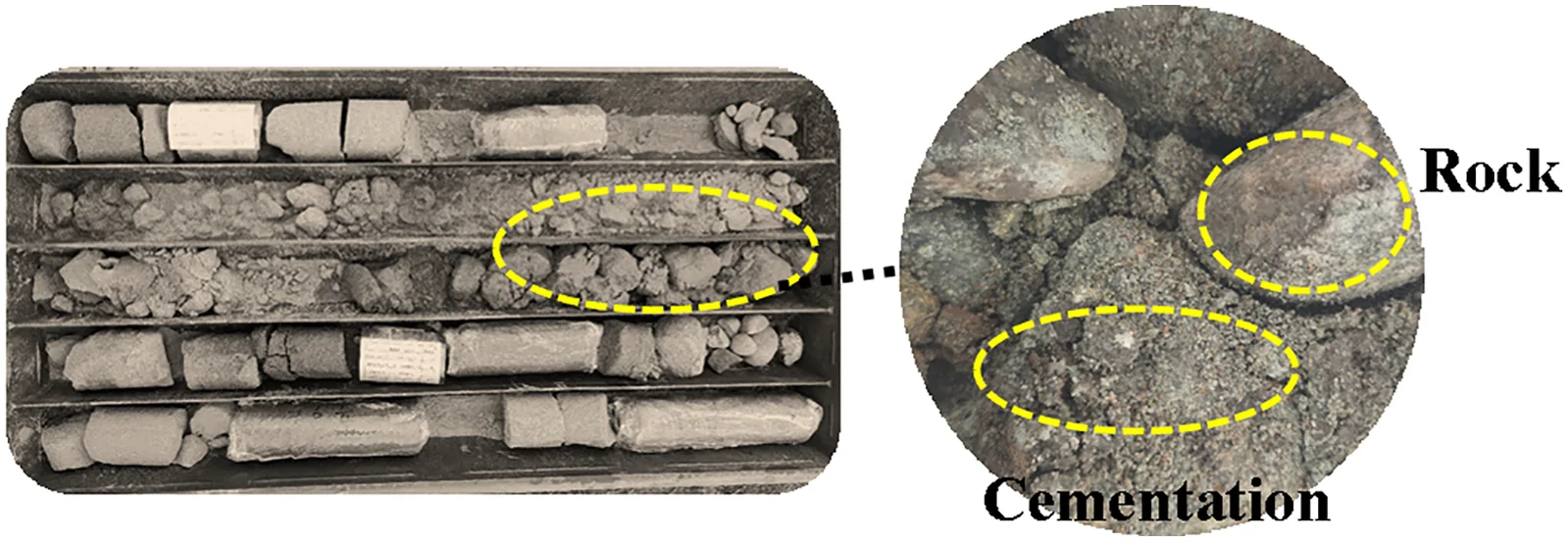
On-site sampling of sandstone.

The overlying aquifer of the main coal seam is not only an important ore-bearing and fluid migration medium in the process of in-situ leaching of uranium, but also a key aquifer that needs key protection and prevention and control in the process of coal mining. Under the influence of factors such as poor gradation of weakly cemented sandstone particles, low cementation strength, and easy softening in water, secondary pore fissure expansion and overburden structure damage are more likely to occur under mining disturbance, increasing the risk of aquifer disturbance infiltration and water channel formation. Under the dual requirements of coal-uranium co-mining and aquifer protection, it is necessary to develop grouting materials suitable for weakly cemented strata, improve the mechanical properties and impermeability of weakly cemented sandstone, and improve the integrity and continuity of key aquifuge. It lays a foundation for improving the grouting efficiency of overlying strata in coal-uranium mining area, improving the effect of fracture plugging, realizing aquifer protection, thickening of aquifuge and safe mining of working face [29].

### Experimental materials and pretreatment methods

The test materials include sandstone samples, coal gangue, polyvinyl alcohol (PVA) and polycarboxylate superplasticizer, and their appearance characteristics are shown in Fig 2.

**Fig 2:**
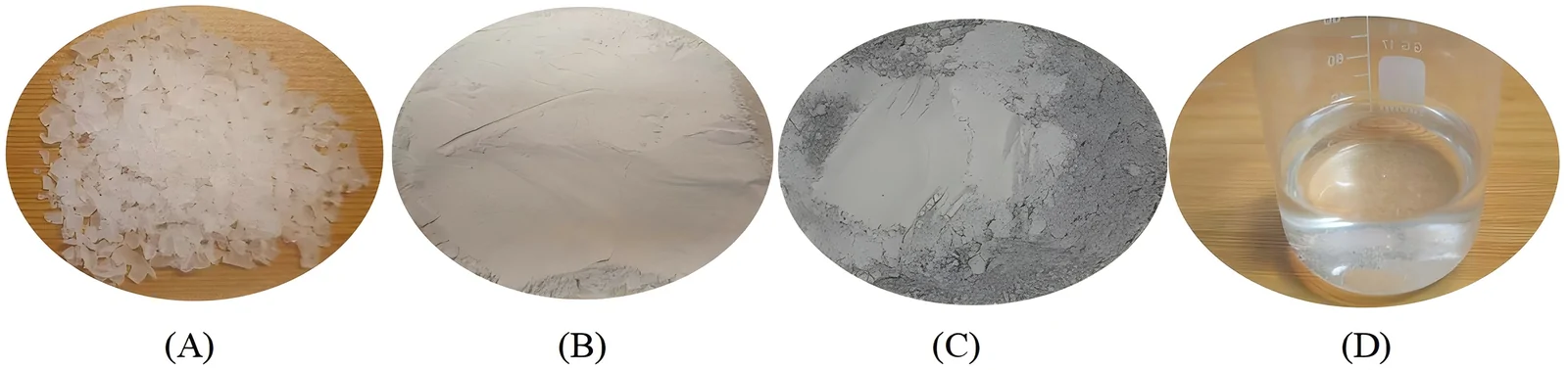
Raw materials used for grout preparation. (A)PVA; (B)Coal gangue; (C)Cement; (D); Polycarboxylate.

#### (1) PVA.

The PVA used in this experiment is a cold water soluble PVA solid particle produced by China Tianjin Zhonglian Chemical Reagent Co.,Ltd., which belongs to a water-soluble polymer with a chemical formula of [-CH_2_CHOH-]_n_. PVA can change the viscosity of the slurry by adjusting the degree of polymerization and hydrolysis [15]. The related chemical parameters are shown in Table 1.

**Table 1:** Technical properties of PVA.

Purity	Residue	Alcoholysis degree/Mol%	Volatile matter	pH Value	Viscosity/mPa·s
98.0	0.5	98.0	5.0	5.0-7.0	54.0-66.0

In order to ensure the consistency of the preparation process of PVA aqueous solution and reduce the influence of different dissolution conditions on the rheological properties and stability of the slurry, all PVA aqueous solutions in the experiment were prepared by a unified process. PVA powder was weighed according to the design ratio, and deionized water (Fig 3a) was slowly added at a ratio of 10:1 at room temperature, and it was fully dispersed by mechanical stirring at 60 rpm to prevent the powder from forming agglomerates after rapid water absorption. Subsequently, the system was heated to 85 °C and stirred continuously for 3–4 h to promote the complete dissolution of PVA particles. After the solution is transparent and uniform as a whole, and no undissolved particles or gel clumps are visible to the naked eye, stop heating and naturally cool to room temperature (Fig 3b).

**Fig 3:**
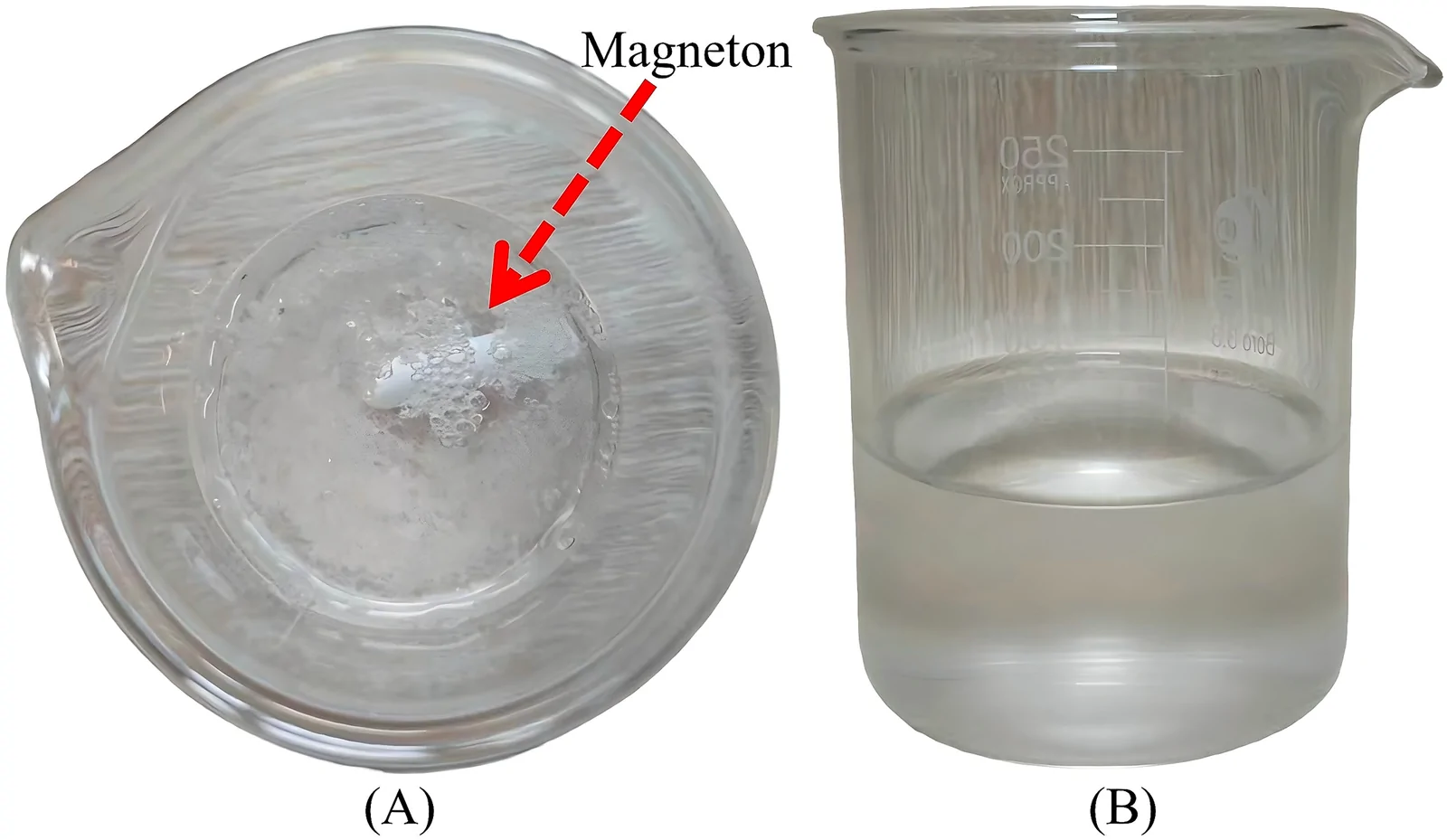
Preparation process and dissolution state of PVA aqueous solution. (A)Before the experiment; (B)After the experiment.

#### (2) Coal gangue(CG).

As shown in Table 2, the main components of CG samples are SiO2 and Al2O3, which are typical aluminosilicate minerals. The high-silica aluminum skeleton can effectively improve the structural stability of the material and is suitable as a raw material for building materials and industrial fillers. Before the experiment, CG needs to be finely ground to 300 mesh. After the particle size is reduced, it is beneficial to increase the specific surface area and enhance its dispersion in the cement-based slurry. The pore structure of the slurry can be improved by the filling effect of fine particles, and the internal connectivity porosity of the hardened body can be reduced.

**Table 2:** XRF composition analysis results of coal gangue.

Ingredients	Na_2_O	MgO	Al_2_O_3_	SiO_2_	P_2_O_5_	SO_3_	Cl	K_2_O	CaO	TiO_2_	Other
Content	0.644	1.766	26.088	60.811	0.055	0.597	0.07	3.959	0.487	1.209	4.314%

#### (3) Cement.

In this experiment, ordinary Portland cement P.O 42.5 was selected as the main cementitious material. The cement is made of natural minerals such as limestone and clay as the main raw materials, which are calcined at high temperature to form Portland cement clinker, and then mixed with an appropriate amount of gypsum and ground. Its main performance indicators meet the relevant requirements of ordinary Portland cement: initial setting time is not less than 45 min, final setting time is not more than 10 h.

#### (4) Polycarboxylate superplasticizer.

The water reducing agent used in this experiment is the standard polycarboxylate high performance water reducing agent produced by Sichuan Dongrun Baisheng New Material Co.,Ltd. According to the product inspection report, the water reduction rate is not less than 25%, and the solid content is 20%, which has strong dispersion water reduction ability and good slurry fluidity regulation effect.

#### (5) Sandstone fragments.

The sandstone fragments of the experimental configuration of the grout-sandstone complex were taken from the Tarangaole coal mine. In order to clarify the microscopic composition and structure of weakly cemented sandstone, XRD, particle size distribution and SEM electron microscope tests were carried out on rock samples in the early stage. As shown in Fig 4, sandstone is mainly composed of quartz and clay minerals. Among them, the quartz content is high, about 53.7%, and the clay mineral content is about 23.4%. It is an important mineral matrix that affects rock water sensitivity, cementation stability and interface action. Sandstone clay minerals are dominated by smectite minerals, about 52% of smectite, about 30% of kaolinite, about 12% of chlorite, and about 6% of illite. Montmorillonite minerals have strong hydrophilicity and expansibility, and are prone to water absorption expansion and particle softening after encountering water. The results of Fig 5a-5d electron microscopy show that the sandstone mineral particles are loosely arranged, the clay mineral filling is discontinuous, and there are obvious intergranular pores, edge pores and microcracks between the particles. The particle size is mainly concentrated in the range of 450 ± 50 μm, and the gradation is relatively concentrated and the connectivity is good.

**Fig 4:**
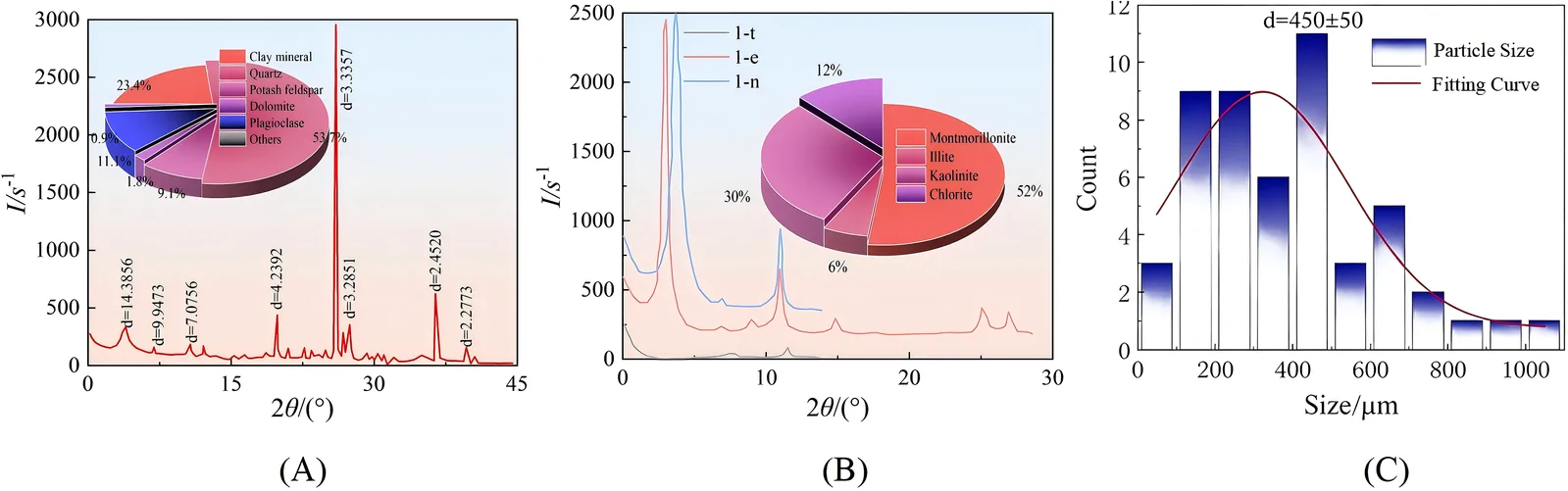
Sandstone XRD and particle size analysis results. (A) whole rock non-clay mineral composition analysis of sandstone; (B) clay mineral composition analysis of sandstone; (C) particle size analysis.

**Fig 5:**
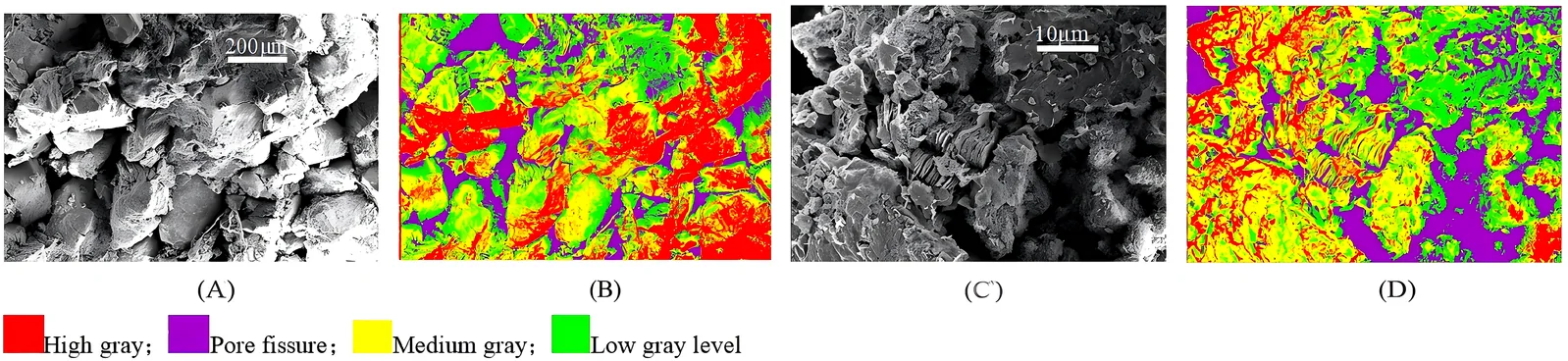
SEM analysis results. (A) Magnification 248; (B) Image processing; (C) Magnification 6840; (D) Image processing.

### Experimental design

As shown in Fig 6, in order to analyze the variation law of micro structure and strength characteristics of slurry rock composite, experiments were carried out from the aspects of material composition, ratio parameters and grouting consolidation effect. Polyvinyl alcohol can regulate the slurry performance and the cementation effect of the slurry-rock interface. The flexible film-like structure formed during the hardening process is beneficial to improve the toughness of the complex, inhibit the development of shrinkage micro cracks, and improve the overall stability of the grout-sandstone complex. Three kinds of PVA content of 0.3%, 0.6% and 1.0% are set. The content of PVA is calculated by formula 1 based on the total mass of cementitious material composed of cement and coal gangue. Among them, 0.3%, 0.6% and 1.0% represent the addition of 0.3 g, 0.6 g and 1.0 g PVA aqueous solution per 100 g cementitious material, respectively. Formula. 1 is the calculation equation:


$$\begin{array}{c} PVA_{\text{dosage}}=\frac{m_{pva}}{m_{\text{cement}}+m_{\text{CG}}}*100\% \end{array}$$ (1)

**Fig 6:**
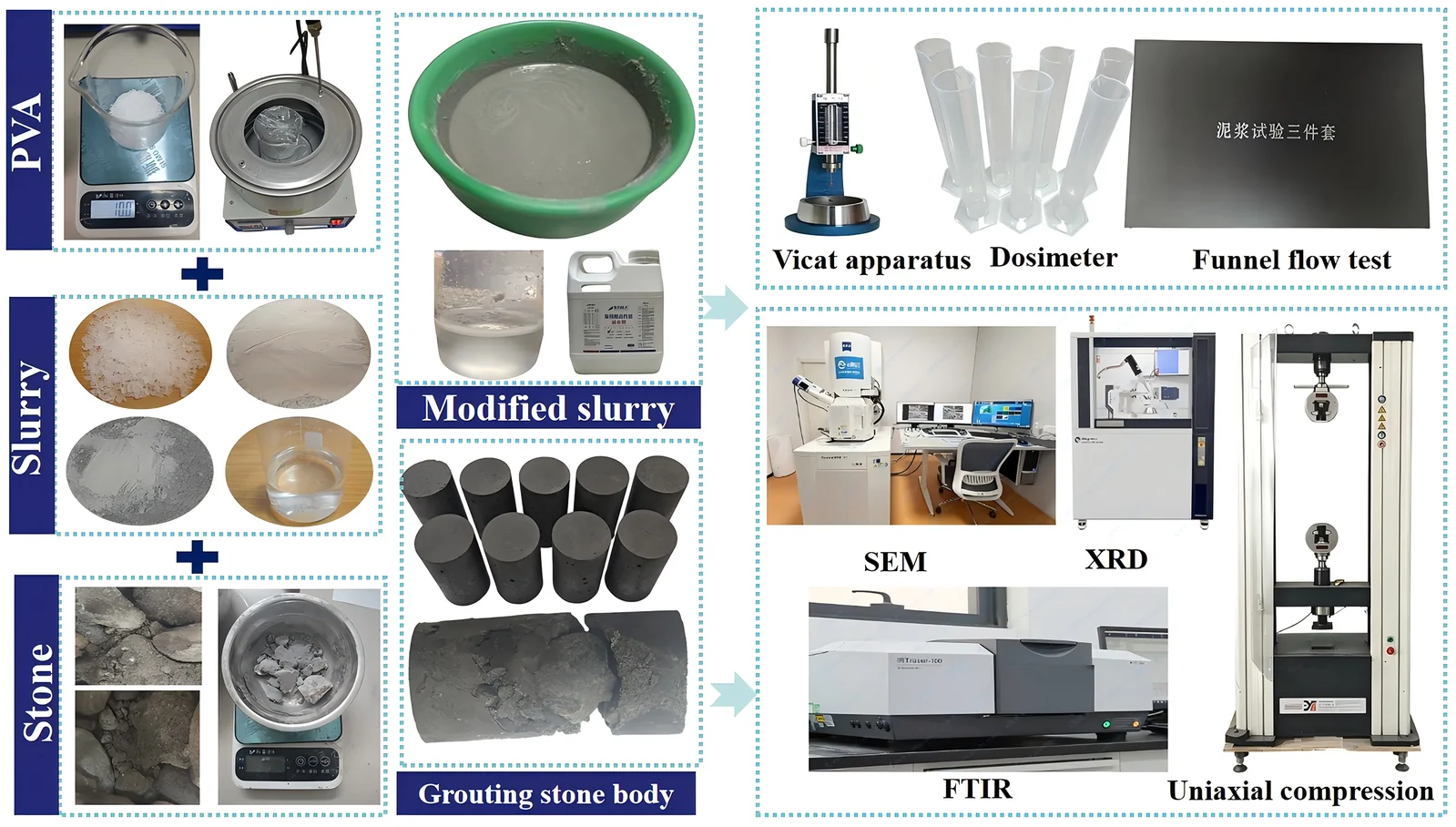
Experimental test flow chart.

Where *m*_*pva*_ is the mass of PVA solution, *m*_*cenment*_ is the mass of cement, and *m*_CG_ is the mass of coal gangue.

Adding an appropriate amount of CG powder into the slurry can use its fine particles to fill the pores inside the slurry, improve the pore structure of the composite, and improve the compactness and impermeability of the slurry-rock composite through hydration reaction. At the same time, this parameter also affects the economy of grouting materials and the level of resource utilization of coal based solid waste. Based on the literature [23], the influence of solid waste content of 60% on the mechanical properties and impermeability of slurry rock composite is analyzed. Through the single factor method, the influence of different mix ratios on the performance of the slurry was analyzed. Water-cement ratio is a key factor affecting slurry fluidity. When the water-cement ratio is too small, the setting property of cement slurry is poor, and segregation and stratification are easy to occur. Excessive water-cement ratio will lead to low slurry strength and high bleeding rate, which will affect grouting compactness and long-term stability. According to the literature [15], the experiment was carried out according to the water-cement mass ratio of 0.3 and the proportion of polycarboxylate superplasticizer of 0.3%. The PVA solution was prepared by pre-dissolution with partially mixed water, and the water used for preparing the PVA solution was included in the total mixed water volume. The experimental scheme is shown in Table 3.

**Table 3:** Mix proportions of the grout specimens.

Number	Cement	Solid waste replacement rate	PVA content/% (by total binder mass)	Water cement ratio
A1	100%	–	–	30%
A2	40%	60%	–	30%
A3	40%	60%	0.3%	30%
A4	40%	60%	0.6%	30%
A5	40%	60%	1.0%	30%

PVA dosage is defined as the mass of PVA relative to the total mass of cement and coal gangue.

### Performance test

In order to ensure the reliability and repeatability of the test results, three parallel tests were conducted for each mixture in the setting-time, bleeding, fluidity, and uniaxial compression tests under identical environmental conditions and operating procedures.

(1) Setting time: refer to GB/T1346-2011 ‘Cement standard consistency water consumption, setting time, stability test method,’ the slurry is molded, scraped and covered, and the initial and final setting time of the slurry is measured by the Vicat instrument.
(2) Bleeding: The slurry was poured into a measuring cylinder with a height of 100 mm. The upper end was sealed with plastic film, and then placed in a constant temperature curing box. The height of the clear water on the surface of the slurry was read every 0.5 h. The percentage value of the total height of water precipitation and the total height of the slurry after standing for 2 hours is called the bleeding rate, as shown in Formual (2):

$$\begin{array}{c} B=\frac{h_{\text{w}}}{100}*100\% \end{array}$$ (2)

Where *B* is the bleeding rate, %; *h*_w_ is the total height of 2h precipitation, mm.

(3) Fluidity: A truncated conical die with a height of 60 mm, an inner diameter of 36 mm at the upper mouth and an inner diameter of 60 mm at the lower mouth was adopted and placed in the center of the horizontal glass plate. The uniformly stirred slurry is quickly injected into the truncated cone mold and flattened, and then the circular mold is lifted smoothly along the vertical direction, so that the slurry can expand freely without disturbance. After the slurry stops flowing, the maximum expansion diameters in two mutually perpendicular directions are measured respectively, and the average value is taken as the slurry fluidity [30].
(4) Microscopic analysis: The mineral composition and content were analyzed by SEM, X-ray diffraction (XRD) and internal standard method. Fourier transform infrared spectroscopy (FTIR) was used to analyze the relative content of hydration products of surface functional groups.

## Result

Table 4 lists the mixture quantities used in the setting-time tests, and Fig 7. presents the results. The initial setting time and final setting time of single cement slurry A1 were 316.67 ± 7.64 min and 371.67 ± 5.77 min, respectively. When the CG replacement rate was 60%, the initial setting time of group A2 increased to 326.67 ± 2.89 min, which was 10.00 min longer than that of group A1, with an increase of about 3.16%. The final setting time increased to 420.00 ± 8.66 min, which was 48.33 min longer than that of A1, with an increase of about 13.00%. It shows that the slurry condensation process is delayed after high proportion of CG substitution, and the influence on the final condensation stage is more obvious [31].

**Table 4:** Mixture quantities used in the setting-time test.

Number	Water(g)	Binder mass(g)	PVA content (%)
A1	150	500	
A2	150	500	
A3	150	500	0.30%
A4	150	500	0.60%
A5	150	500	1.00%

**Fig 7:**
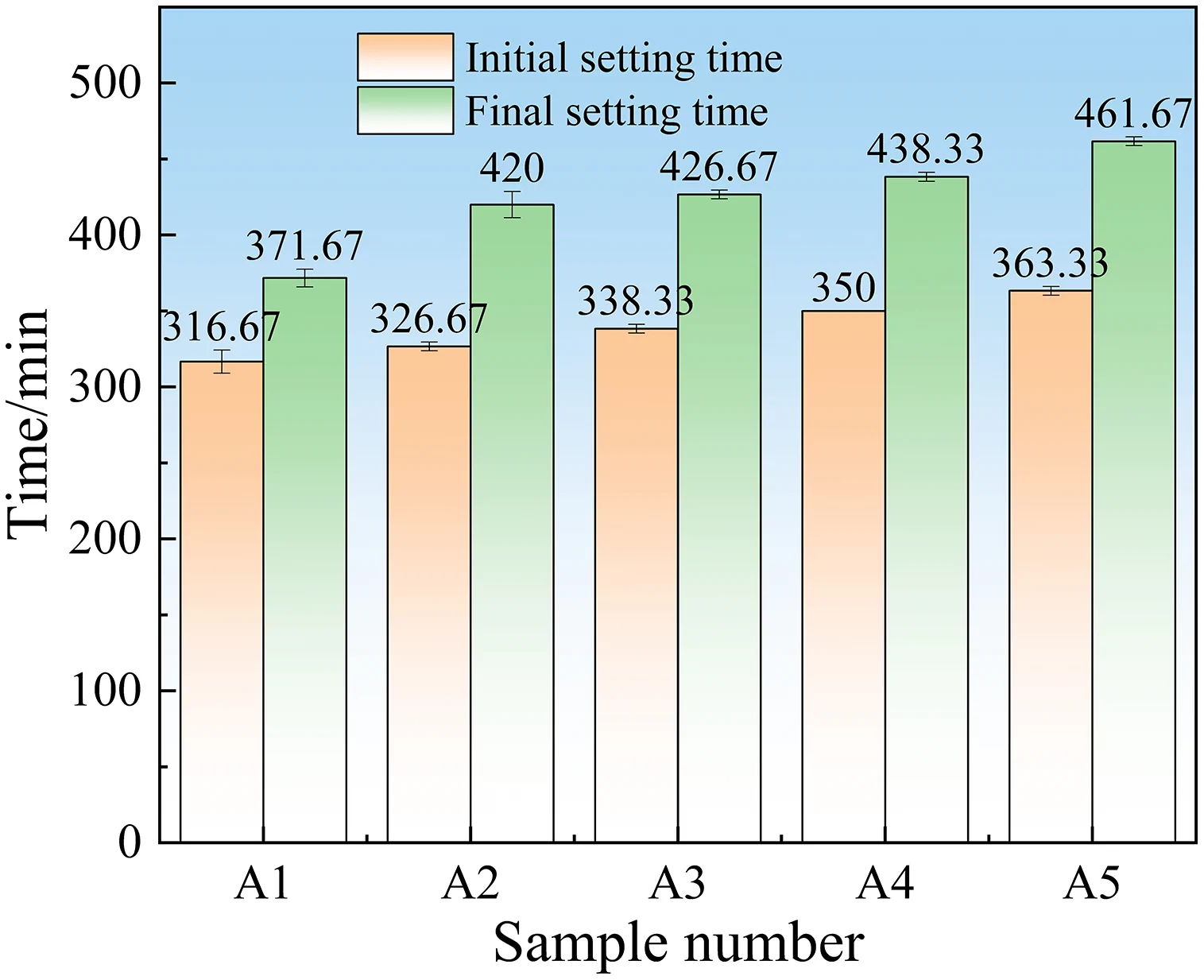
Test results of setting time.

With the increase of PVA content from 0.30% to 1.00%, the initial setting time of A3, A4 and A5 groups was 338.33 ± 2.89, 350.00 ± 0.00 and 363.33 ± 2.89 min, respectively, and the final setting time was 426.67 ± 2.89, 438.33 ± 2.89 and 461.67 ± 2.89 min, respectively. Compared with the A2 group without PVA, the initial setting time of A3, A4 and A5 groups was prolonged by 11.66,23.33 and 36.66 min, respectively, with an increase of 3.57%, 7.14% and 11.22%, respectively. The final setting time was prolonged by 6.67, 18.33 and 41.67 min, respectively, with an increase of 1.59%, 4.36% and 9.92%, respectively. Compared with group A1, the initial setting time and final setting time of group A5 were prolonged by 46.66 and 90.00 min, respectively.

The above results show that both CG substitution and PVA incorporation have a certain delay effect on the slurry condensation process. After CG replaces cement, the early active components such as C_3_S, C_2_S and C_3_A in the system are relatively reduced, which reduces the rate of early hydration reaction. After the addition of PVA, the cohesion and water retention characteristics of the slurry system change, which may also affect the particle migration and the formation process of early hydration products, thus further prolonging the setting time of the slurry.

As shown in Fig 8, the bleeding rate of each group was characterized by the average value of three parallel experiments. The bleeding rate of single cement slurry A1 was 1.163 ± 0.015%. When the CG replacement rate was 60%, the bleeding rate of A2 group increased to 2.473 ± 0.015%, which was about 112.61% higher than that of A1. This indicates that the slurry stability is reduced after high proportion of CG substitution. The reason may be related to the relatively low hydration activity of CG.After the decrease of cement content, the formation of early hydration products is affected to a certain extent, and the binding capacity of the system to free water is weakened, thus increasing the bleeding tendency.

**Fig 8:**
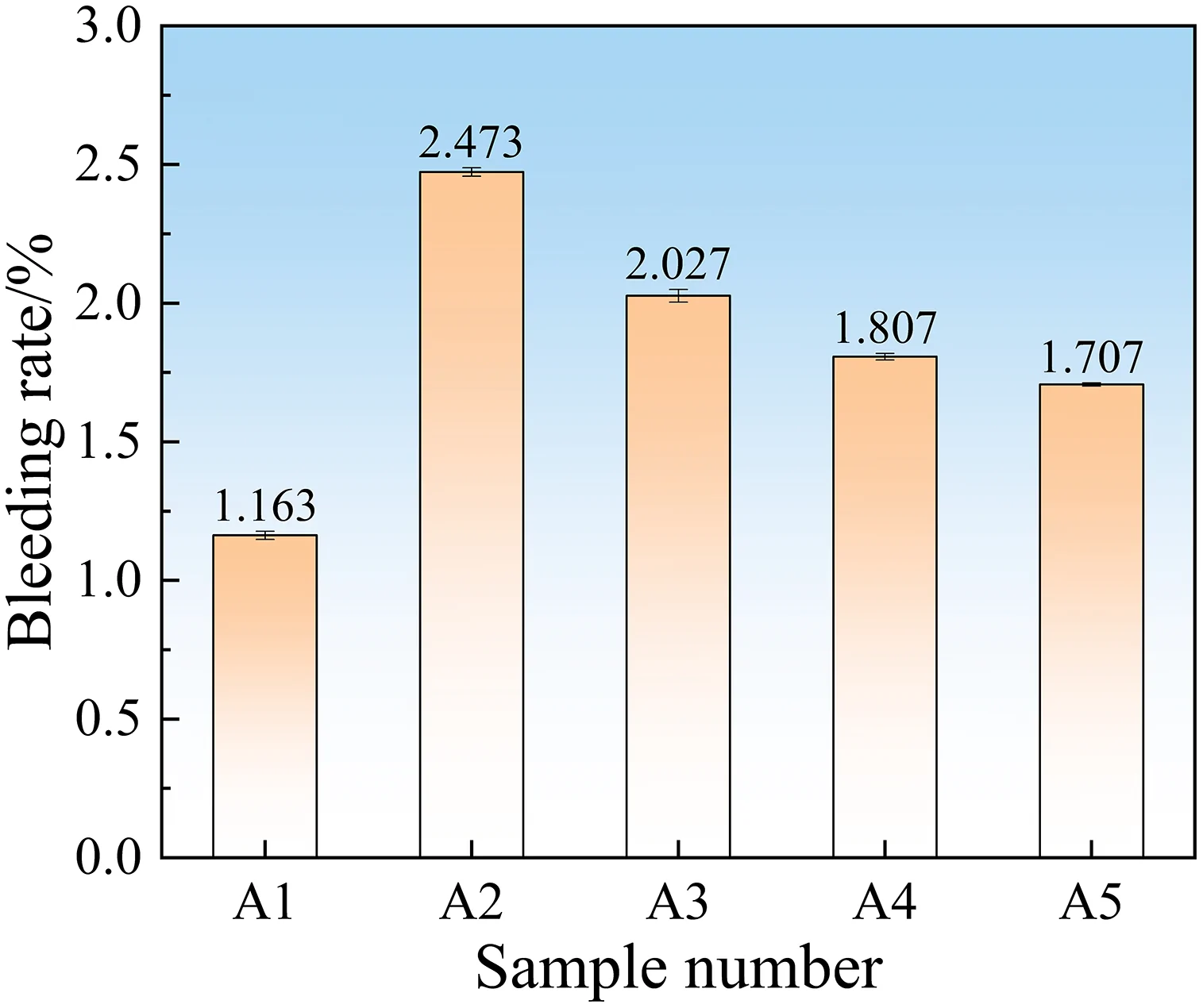
Bleeding rate test results.

After the incorporation of PVA, the bleeding rate of the slurry generally showed a downward trend. The bleeding rates of A3, A4 and A5 groups were 2.027 ± 0.023%, 1.807 ± 0.012% and 1.707 ± 0.006%, respectively, which were 18.06%, 26.95% and 31.00% lower than those of A2 group. From the changes of adjacent PVA groups, A3 to A4 decreased by 10.86%, A4 to A5 decreased by 5.54%, indicating that with the increase of PVA content, the slurry bleeding rate continued to decrease, but the improvement of high content stage was reduced.

The effect of PVA on bleeding performance may be mainly related to its hydrophilicity, water retention and regulation of slurry cohesion. The PVA molecular chain contains more hydroxyl groups, which is beneficial to improve the water retention ability of the system. At the same time, the addition of PVA can change the suspension and sedimentation state of particles in the slurry, thus reducing the degree of free water precipitation.

Table 5 is the amount of liquidity experiment. As shown in Fig 9, the fluidity of single cement slurry A1 is 223.00 ± 2.00 mm; when the CG replacement rate reached 60%, the fluidity of group A2 decreased to 152.67 ± 1.53 mm, which was 70.33 mm lower than that of group A1, with a decrease of about 31.54%. This shows that the high proportion of CG substitution significantly reduces the fluidity of the slurry, which may be related to the rough surface of the coal gangue particles, the large specific surface area and the adsorption of free water [32].

**Table 5:** Mixture quantities used in the fluidity test.

Number	Water consumption(g)	Binder mass(g)
A1	87	300
A2	87	300
A3	87	300
A4	87	300
A5	87	300

**Fig 9:**
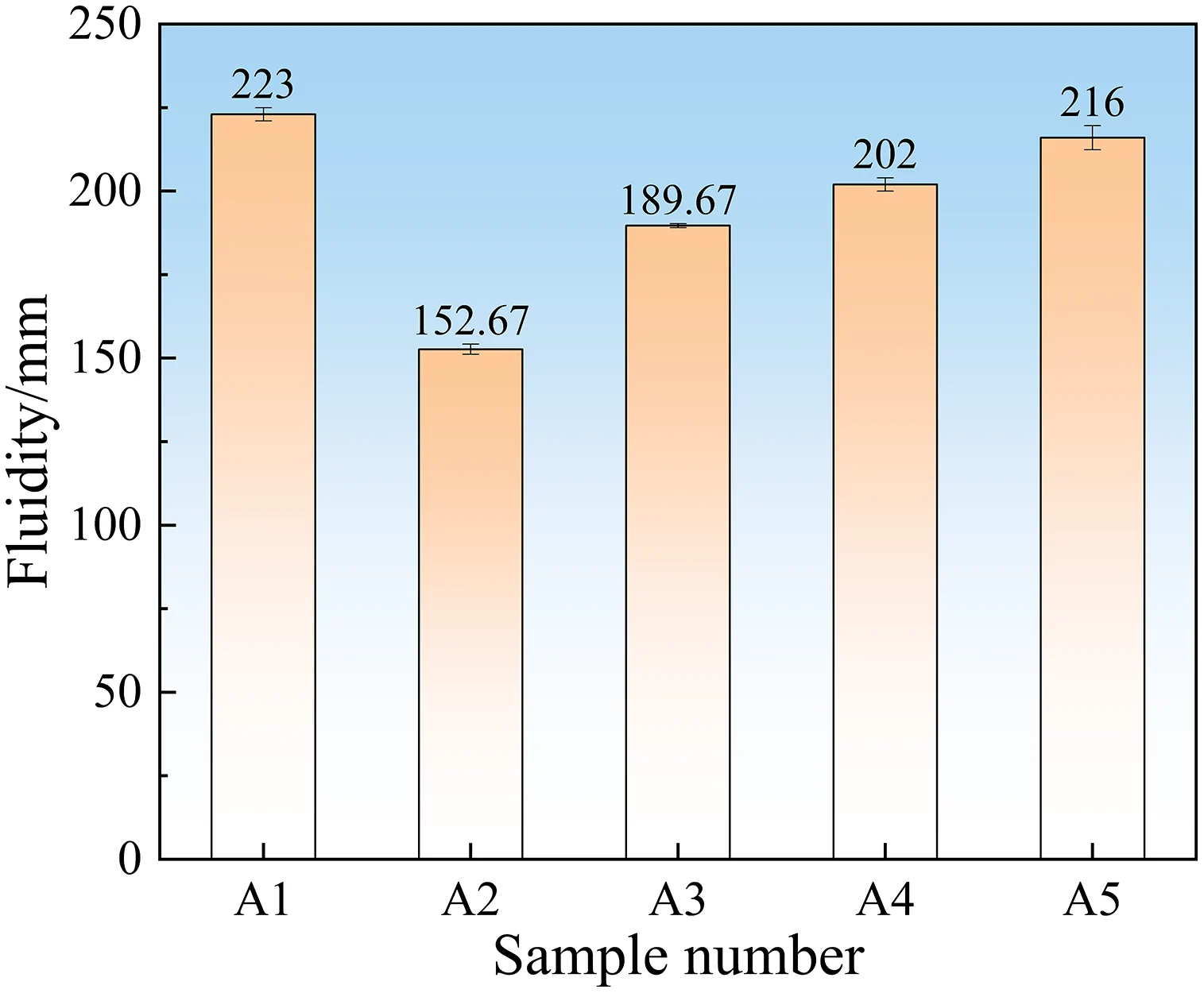
Fluidity test results.

After adding PVA, the fluidity of CG-cement slurry is generally improved. The fluidity of A3, A4 and A5 groups was 189.67 ± 0.58, 202.00 ± 2.00 and 216.00 ± 3.61 mm, respectively, which was 37.00, 49.33 and 63.33 mm higher than that of A2 group, and the increase was 24.24%, 32.31% and 41.48%, respectively. From the perspective of the change of adjacent ratio, the fluidity of A2 to A3 groups increased the most, from 152.67 mm to 189.67 mm, with an increase of 24.24%. A3 to A4 increased by 12.33 mm, an increase of 6.50%; a4 to A5 increased by 14.00 mm, an increase of 6.93%. It is worth noting that although the fluidity of group A5 is close to that of single cement slurry A1, it is still about 3.14% lower than that of A1.

The above results show that PVA can improve the fluidity of high CG content slurry to a certain extent within the tested mix-proportion range. This change may be related to the comprehensive adjustment of PVA on the water retention, particle dispersion state and slurry stability of the system, so that some of the lost fluidity after CG incorporation can be restored.

### Microstructure analysis

The main compounds of cement include Tricalcium silicate (C_3_S), Dicalcium silicate (C_2_S), Tricalcium aluminate (C_3_A), Tetracalcium aluminoferrite (C_4_AF) and Gypsum (CSH_2_). In the process of cement hydration, the following reactions will occur [33]:


$$\begin{array}{c} 2C_{\text{3}}S+6H\to C_{\text{3}}S_{\text{2}}H_{\text{3}}+3CH \end{array}$$ (3)

$$\begin{array}{c} 2C_{\text{2}}S+4H\to C_{\text{3}}S_{\text{2}}H_{\text{3}}+CH \end{array}$$ (4)

$$\begin{array}{c} C_{\text{3}}A+3CSH_{\text{2}}+26H\to C_{\text{6}}AS_{\text{3}}H_{32} \end{array}$$ (5)

The reaction and products of C_3_S, C_2_S and C_3_A with water in cement are the main sources of C-S-H, C-H and AFt in cement system. When CG is introduced into the system as a mineral admixture, the active SiO_2_ and Al_2_O_3_ can react with the C-H generated by cement hydration to generate more C-S-H, C-A-H or C-A-S-H gels. The generalized reaction formula can be expressed as [34]:


$$\begin{array}{c} 2SiO_{\text{2}}+CH+H+aq\to C_{\text{3}}S_{\text{2}}H_{\text{3}}+3CH \end{array}$$ (6)

$$\begin{array}{c} C_{\text{3}}AS_{\text{3}}H_{32}+2C_{\text{4}}AH_{13}=3\left(C_{\text{3}}ASH_{12}\right)+2CH+20H \end{array}$$ (7)

As shown in Fig 10, the main diffraction peaks of the XRD patterns of A1-A5 specimens are concentrated near 2*θ* = 18°, 26° ~ 35°, 45° ~ 55° and 60°, indicating that the main controlling mineral phases of different proportions of slurry are basically the same. A1 has a strong peak at 2*θ* ≈ 18°, corresponding to the characteristic peak of Ca (OH)_2_, indicating that the crystal phase has high crystallinity and dominant content. At the same time, multiple sets of overlapping peaks of C_3_S, calcite and silicate minerals can be seen in the range of 2*θ* ≈ 29° ~ 34°, and the peak shape is narrow, indicating that the crystalline hydration products and residual clinker minerals in the single cement system are obvious.

**Fig 10:**
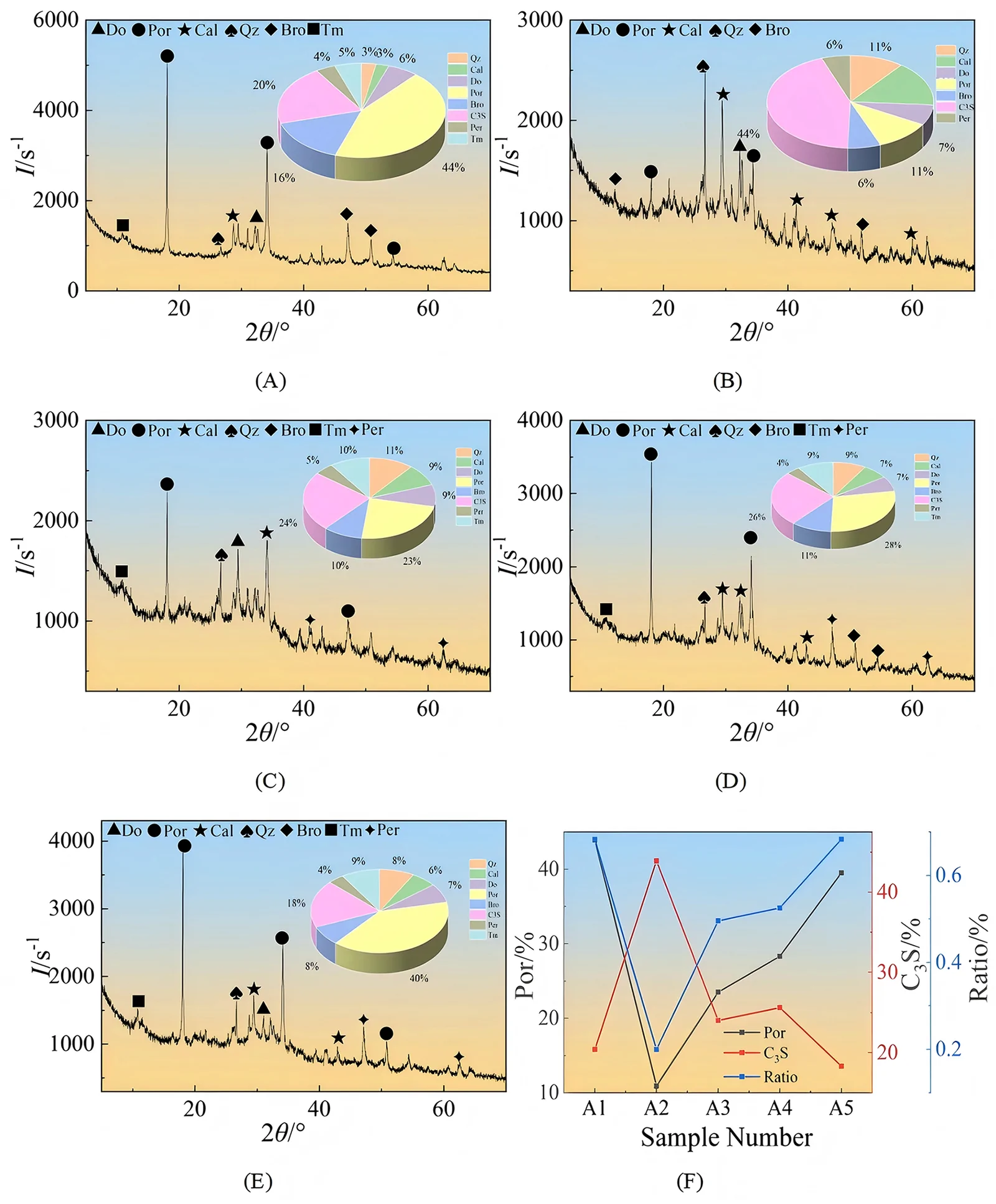
XRD whole rock mineral detection results. (A)A1; (B)A2; (C)A3; (D)A4; (E)A5; (F)*R* value.

After A2 is mixed with 60% coal gangue, the quartz peak near 2θ ≈ 26.6°, the carbonate peak near 29°-31° and the C_3_S related peak near 31°-34° are significantly enhanced, while the Ca(OH)_2_ peak near 18° is significantly weakened. Combined with the pie chart, the proportion of C_3_S increased to 43.90% and Ca(OH)_2_ decreased to 10.95%, indicating that the dominant crystal phase of A2 changed from Ca(OH)_2_ to residual silicate and coal gangue-derived minerals. The content of quartz and calcite in A2 increased to 10.54% and 15.58%, respectively, while the content of portlandite decreased to 10.95%, and the content of tricalcium silicate increased to 43.90%, indicating that the high proportion of coal gangue substitution reduced the hydration activity of the system, so that some cement clinker minerals failed to fully participate in the hydration reaction, resulting in an increase in the unhydrated silicate phase residue.

After adding PVA, the C-H peaks near 2*θ* ≈ 18° and 34° in the A3-A5 spectra gradually recovered and enhanced, and the peak group in the range of 2*θ* ≈ 29° ~ 35° gradually shifted from the sharp residual C_3_S peak to the superposition of crystal peaks. FTIR and electron microscopy analysis showed that in addition to the increase of C-H crystals in the system, the low-crystallization C-S-H gel phase also increased. Among them, A5 has the highest C-H peak intensity near 18°, indicating that PVA is conducive to promoting the conversion of clinker phase to cementitious products. PVA incorporation improves the degree of hydration of the system. The proportion of main crystalline phases in the system changes after the incorporation of PVA, indicating that it has a certain regulatory effect on the hydration reaction process. Compared with A2, the content of Ca(OH)_2_ in A3, A4 and A5 increased to 23.53%, 28.32% and 39.54%, respectively, which were significantly higher than those in A2 without PVA. The corresponding C_3_S residues decreased to 23.95%, 25.60% and 18.34%, respectively, which were significantly lower than 43.90% in group A2.

In order to compare the relative changes between crystalline hydration products and residual clinker phases in different ratio systems, this paper introduces a semi-quantitative index *R* to characterize the proportional relationship between the relative content of C-H and the residual C_3_S:


$$\begin{array}{c} R=\frac{C{\text{a(OH)}}_{\text{2}}}{C_{\text{3}}S+C{\text{a(OH)}}_{\text{2}}} \end{array}$$ (8)

The increase of R value indicates that the relative content of C-H corresponding to unit residual C3S increases, reflecting the enhanced transformation trend of clinker phase to crystalline hydration products. However, since low crystalline or amorphous C-S-H is difficult to be accurately and independently quantified by conventional XRD, and this index does not contain all hydration products, R is only used as an auxiliary semi-quantitative characterization of the hydration reaction trend between different samples. As shown in Fig 10, the R value of A2 group is 0.20, while the R value of A3~ A5 group increases after PVA incorporation, indicating that the relative ratio between C-H and residual C3S changes significantly, and the trend of clinker phase to crystalline hydration products is enhanced. A5 is close to A1 level, indicating that the clinker phase in the system still continues to transform into hydration products after PVA is added.

Fig 11 is the FTIR characteristic spectrum of the five specimens. Among them, the wide absorption band near 3000−3700 cm^-1^ is mainly related to the hydroxyl structure in Ca(OH)_2_; the absorption peak near 1650 cm^-1^ corresponds to the bending vibration of H-O-H. The absorption peak near 1400 cm^-1^ is mainly derived from the asymmetric stretching vibration of CO_3_^2-^ group, which is related to the carbonation of Ca(OH)_2_ and the formation of carbonate phase. The characteristic peak near 1100 cm^-1^ is mainly the stretching vibration of Si-O bond in C-S-H gel, which is an important characteristic peak reflecting the structure of silicate hydration products and coal gangue aluminosilicate. The peak near 850 cm^-1^ can be attributed to the out-of-plane bending vibration of CO_3_^2-^ or the C-O related vibration peak. Through the deconvolution fitting analysis of the 800−1600 cm^-1^ characteristic interval, the quantitative information of the structural evolution of the hydration products was obtained and analyzed with the electron microscope test results. As shown in. Fig 11 and Fig 12, the hydration products of single cement specimens are more obvious, and more flocculent and cluster C-S-H gels can be observed in the SEM images, accompanied by needle-like AFt crystals and flake C-H crystals. In the FTIR spectrum, the Si-O related peak was obvious, the peak position was 989.48 cm^-1^, the main peak of CO_3_^2-^ was located at 1392.61 cm^-1^, and the peak area contribution accounted for 63.1%, indicating that the C-S-H gel structure was more developed.

**Fig 11:**
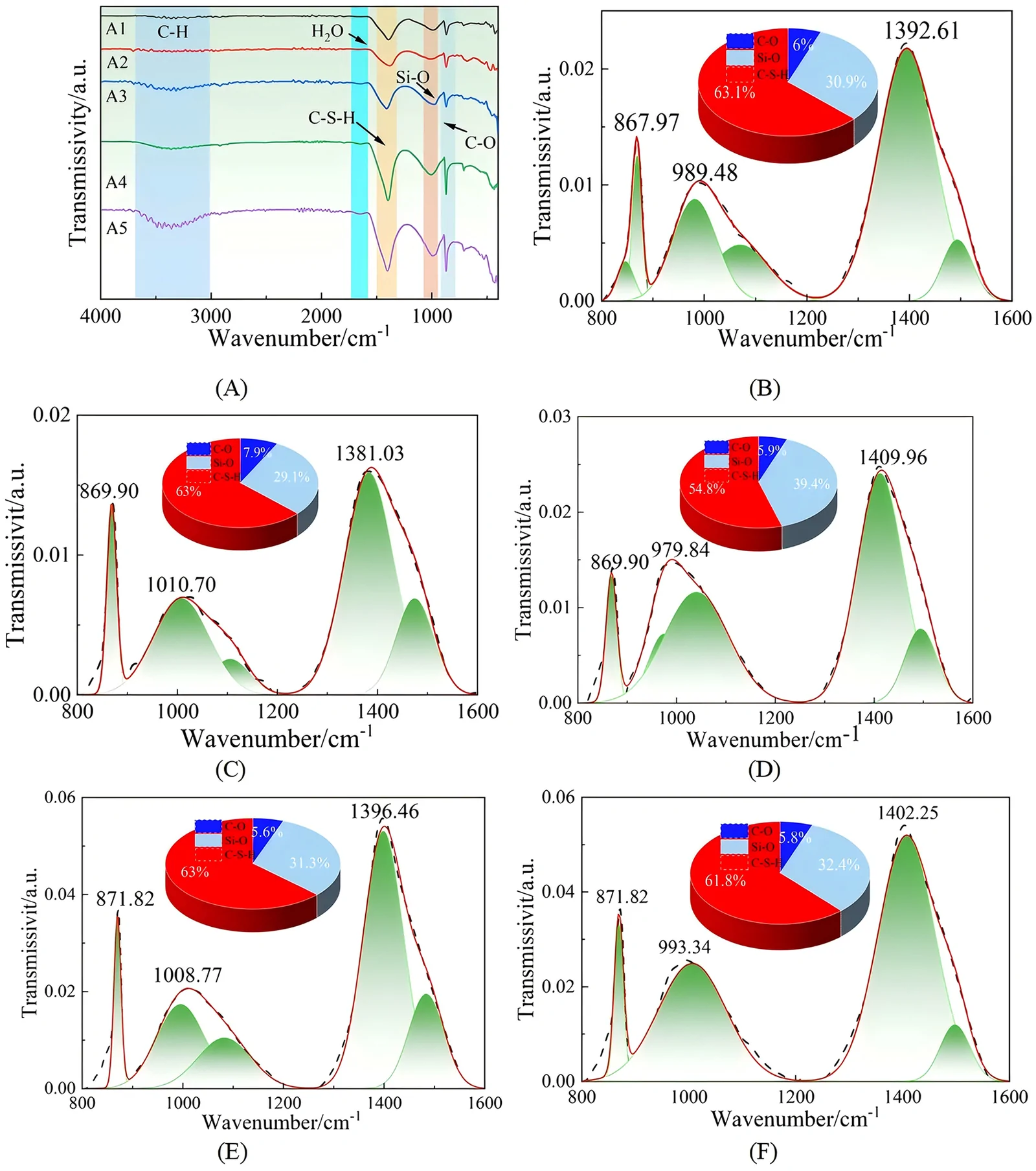
FTIR spectroscopy test results. (A)FTIR characteristic spectrum; (B)A1 deconvolution fitting calculation results; (C)A2 deconvolution fitting calculation results; (D)A3 deconvolution fitting calculation results; (E)A4 deconvolution fitting calculation results; (F)A5 deconvolution fitting calculation results.

**Fig 12:**
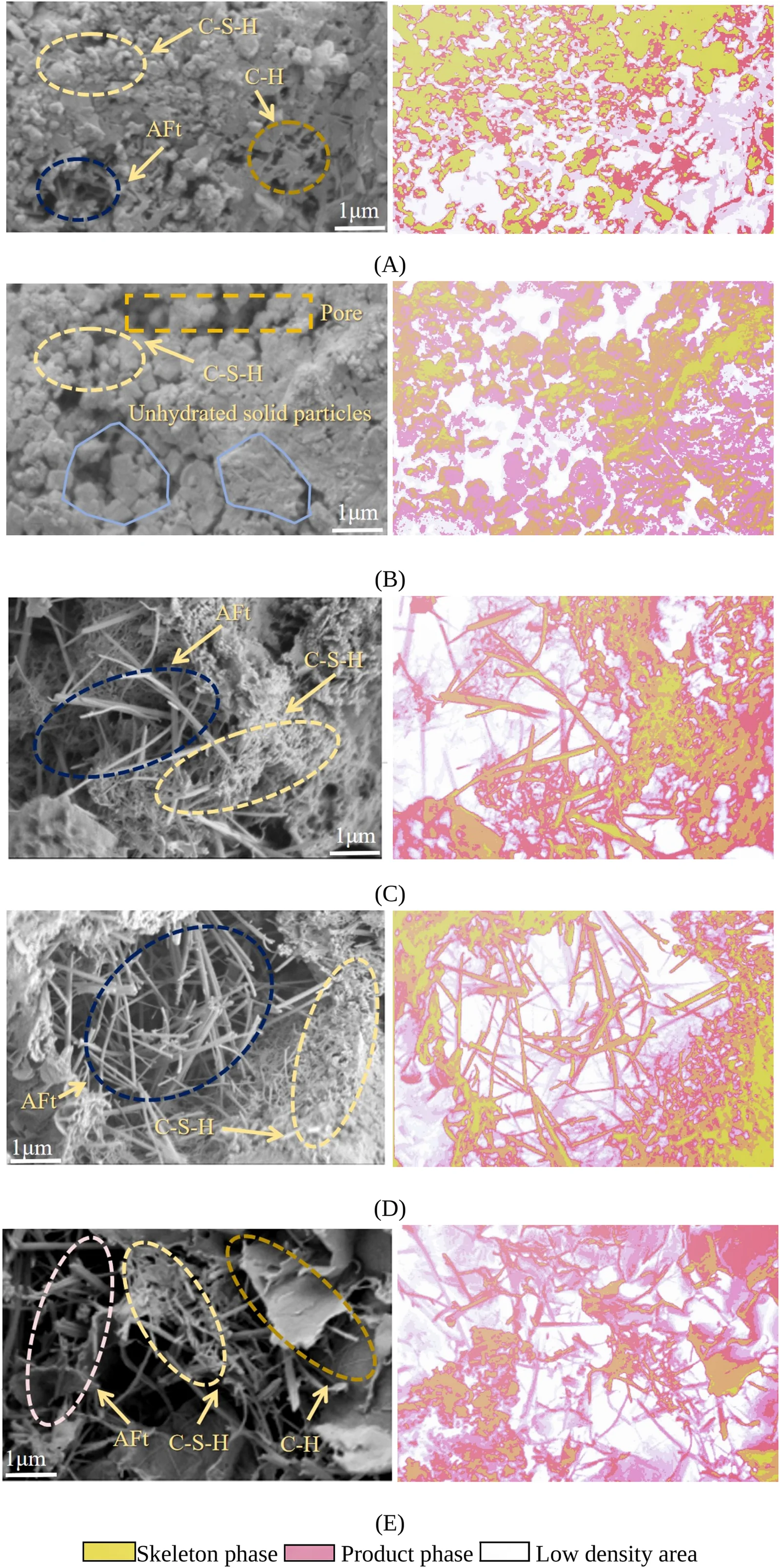
SEM test results. (A) SEM micrograph and pseudo-colored phase map of sample A1; (B) SEM micrograph and pseudo-colored phase map of sample A2; (C) SEM micrograph and pseudo-colored phase map of sample A3; (D) SEM micrograph and pseudo-colored phase map of sample A4; (e) SEM micrograph and pseudo-colored phase map of sample A5.

After the incorporation of coal gangue, the microstructure of the system will be transformed into a synergistic process of secondary pozzolanic reaction between coal gangue and cement. As shown in Fig 11c, the A2 low wave number peak moves from 867.97 cm^-1^ to 869.90 cm^-1^, and the Si-O peak moves from 989.48 cm^-1^ to 1010.70 cm^-1^, indicating that the chemical environment of Si-O bond changes after the introduction of aluminosilicate components in coal gangue. The proportion of low wavenumber peak area of A2 increases, and the proportion of Si-O peak area decreases, indicating that the effective hydration cementitious structure is weakened. The SEM results also show that there are obvious unreacted coal gangue particles in A2, the particle boundary is clear and the surface is rough, and there are obvious interface differences between A2 and the surrounding gel matrix. Because the early activity of coal gangue is lower than that of cement, the formation of early hydration products such as C-S-H and AFt is insufficient, and the continuity of interface cementation is weaker than that of A1.

The PVA molecular chain contains a large number of hydroxyl groups, which can form hydrogen bonds with water molecules, so that part of the free water is converted into adsorbed water or bound water, thereby reducing the bleeding and segregation tendency of the slurry, and maintaining a stable hydration environment around the cement particles and coal gangue particles. Stable water supply is conducive to the continuous hydration of C_3_S and C_2_S to form C-S-H gel, and also provides the necessary conditions for the reaction of C_3_A with gypsum to form AFt. Combined with the development morphology of AFt, C-S-H and C-H structures in Fig 12, the PVA content changed the number, spatial distribution and pore filling mode of slurry hydration products.

When the content of PVA is 0.3%, the appearance of Si-O related structure is promoted, and the contribution of Si-O peak area of A3 increases from 29.1% to 39.4%, an increase of about 35.4%. In the SEM results, C-S-H is mainly attached to the surface of the particles in the form of flocculent and gel. The number of AFt crystals is small, the development length is limited, and there are still pores and loose areas in the local area. Compared with A3, the Si-O peak position of A4 shifted from 979.84 cm^-1^ to 1008.77 cm^-1^, indicating that the silicon-oxygen structure was gradually transformed from a relatively dispersed aluminosilicate or primary gel state to a more stable hydration gel structure. The response of A4 related characteristic peaks was enhanced. Combined with FTIR and SEM results, the composition and spatial distribution of hydration products changed significantly under the condition of 0.6% PVA. SEM results also show that a large number of needle-like AFt crystals in A4 grow interwovenly between pores, and C-S-H gel is further enriched on the surface of the particles and inside the pores to form a more continuous cementation structure.

The proportion of CO_3_^2-^ main peak area in A5 decreased, indicating that there was an upper limit for the promoting effect of PVA on the hydration cementation structure, and the increase of PVA content further enhanced the O-H broad peak. More flaky C-H crystals can also be observed in the SEM images, indicating that the hydration reaction of the system is still ongoing, but excessive C-H crystals are likely to cause local structure coarsening. At the same time, partially hydrolyzed PVA continued to hydrolyze in the strong alkaline pore solution of cement, releasing acetate, and the released acetate complexed with Ca^2+^, delaying the formation of ettringite by C3A and gypsum.

In general, an appropriate amount of PVA can promote the uniform deposition of hydration products and enhance the interface cementation. When the content of PVA is too high, excessive polymer may coat cement and coal gangue particles, hinder the diffusion of water and ions, delay the hydration of cement and cause local weak interface.

## Discussion

As shown in Table 6, in order to further analyze the cementation enhancement effect of PVA modified grouting material on weakly cemented sandstone, 150 g sandstone fragments were initially weighed in each group of tests, and 250 g fresh slurry was added for composite pouring. The total mass of initial feeding was 400 g. After pouring and standard curing for 28 days, the specimens were processed into standard cylinders with a diameter of 50 mm and a height of 100 mm. Three independent parallel specimens were set up for each ratio, and uniaxial compression tests were carried out after curing under the same conditions. In the test, the loading rate was set to 0.005 mm/s, and the axial load and axial deformation data were continuously collected during the loading process until the specimen was obviously damaged. The maximum axial stress on each stress–strain curve was defined as the peak stress. Failure was considered to occur when the load-bearing capacity decreased markedly after the peak stress, accompanied by macroscopic crack propagation or overall instability.

**Table 6:** Uniaxial compression test results.

Groups	Group 1	Group 2	Group 3	Results
B0	4.90	4.99	4.72	4.87 ± 0.14
B1	5.38	5.28	5.39	5.35 ± 0.06
B2	6.06	5.94	6.11	6.04 ± 0.09
B3	5.13	5.03	5.21	5.12 ± 0.09

The peak stress of grout-sandstone complex in B0, B1, B2 and B3 groups was 4.87 ± 0.14, 5.35 ± 0.06, 6.04 ± 0.09 and 5.12 ± 0.09 MPa, respectively, and the coefficient of variation was 2.82%, 1.14%, 1.45% and 1.76%, respectively. Compared with the B0 group without PVA, an appropriate amount of PVA can improve the peak bearing capacity of the composite, and the average peak stress of the 0.6% PVA group is the highest. When the PVA content is further increased to 1.0%, the peak stress decreases. This shows that PVA has a certain dosage dependence on the mechanical response of the slurry-rock composite. Within the test range of this paper, the 0.6% group shows relatively better mechanical properties.

In order to further analyze the microstructure characteristics of the slurry-rock interface, SEM observation was carried out in this paper, and the first group of representative microstructure results were selected for display and analysis. In Table 7, 380.9 ~ 389.8 g is the measured mass of the standard cylindrical specimen after processing. In the process of forming and processing, there are residual molds and mixing utensils, free water loss in the process of end face trimming and curing.

**Table 7:** Mix proportions and specimen information.

Number	Sand and gravel fragments(g)	Slurry(g)	Solid waste replacement rate(%)	PVA dosage (%)	water cement ratio	Measured mass of the Φ50 mm × 100 mm specimen after 28 d curing/g
B0	150	250	60	0	0.3	387.2
B1	150	250	60	0.3	0.3	380.9
B2	150	250	60	0.6	0.3	389.8
B3	150	250	60	1.0	0.3	383.4

As shown in Fig 13, the peak stresses of B0, B1, B2 and B3 specimens are 4.90, 5.38, 6.06 and 5.13 MPa, respectively. Based on the B0 group without PVA, after adding 0.3%, 0.6% and 1.0% PVA, the peak stress of the specimen increased by about 9.8%, 23.7% and 4.6%, respectively, indicating that the introduction of PVA can generally improve the bearing capacity of the slurry-rock composite, but its enhancement effect has obvious dosage dependence. When the PVA content was further increased to 1.0%, the peak stress of B3 decreased to 5.13 MPa, which was about 4.8% lower than that of B1, indicating that the decrease of peak strength and elastic modulus of 1.0% PVA group was not only controlled by the lack of overall hydration, but also related to the local polymer thick film coating, C-H crystal enrichment, interface structure non-uniformity and the increase of flexible phase ratio. The peak strain gradually increases with the increase of PVA content. Compared with B1, the peak strain of B2 and B3 increases by about 6.4% and 7.3%, respectively, indicating that the higher PVA content makes the composite change from rigid cementation structure to flexible cementation structure, and can accommodate greater axial deformation before failure. Although the peak strength of the B3 specimen decreased, the peak strain remained at a high level, indicating that the higher PVA content enhanced the flexibility and ductility of the composite, making the specimen more resistant to deformation. The elastic modulus of B0, B1, B2 and B3 are 2.38,2.45,2.59 and 2.17 GPa, respectively. Compared with B0 group, the elastic modulus of B1 and B2 groups increased by about 2.8% and 8.8%, respectively. B3 decreased by about 11.4% compared with B1, indicating that the PVA film structure has a certain flexibility and plays a role of cementation buffer at the particle contact interface.

**Fig 13:**
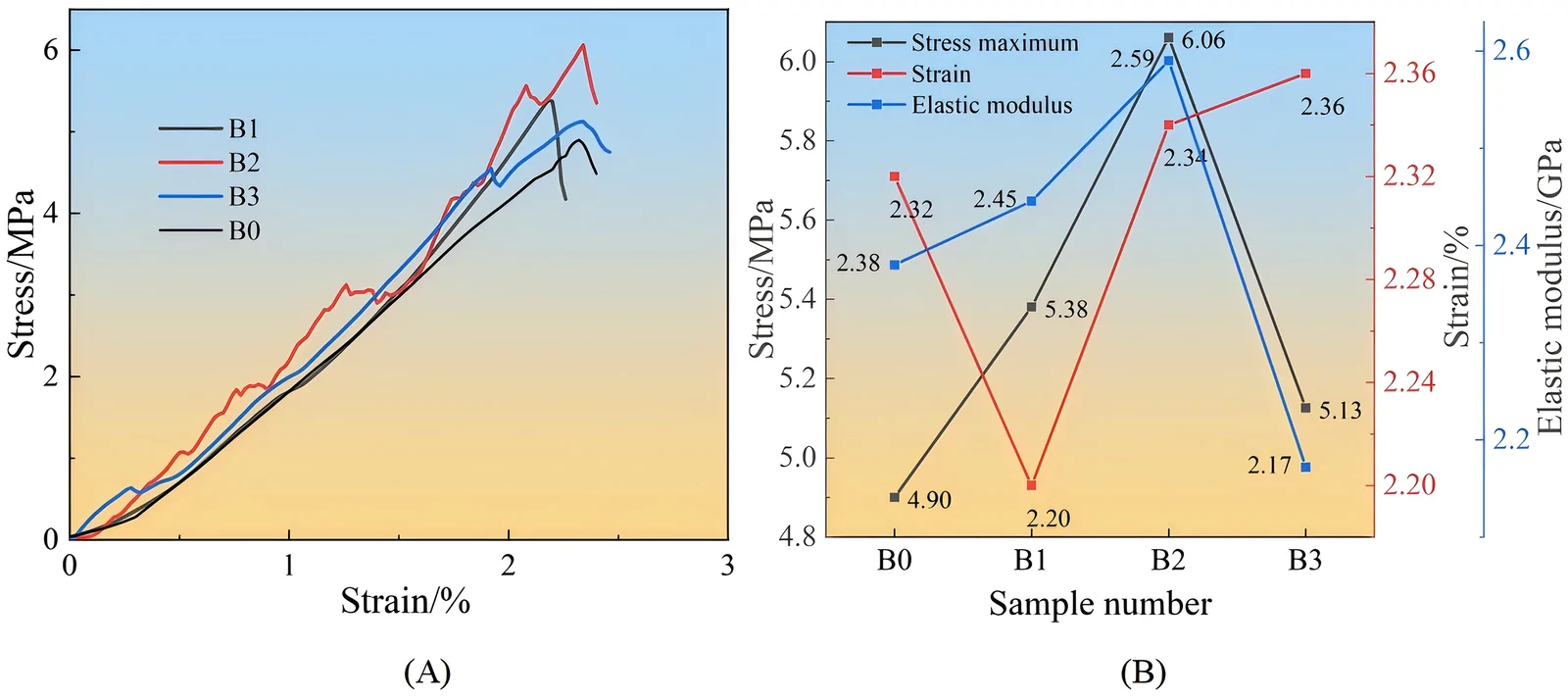
Uniaxial compression test results. (A) Stress-strain curve; (B) Characteristics of mechanical parameters.

On the whole, the B2 specimen showed better mechanical properties, and its peak stress was the highest, which was 6.06 MPa. The peak strain and elastic modulus were higher than those of B1, indicating that 0.6% PVA content can achieve a better balance between strength improvement and deformation coordination. Therefore, from the perspective of the strength and toughness of the solid waste-based slurry cemented sand and gravel block, the PVA content should be controlled within the appropriate range. In this test, the effect of 0.6% PVA content is better.

Fig 14 is the SEM electron microscope results of sandstone composite under different PVA content. Compared with the microstructure of the existing superfine cement grouting complex, the slurry-rock interface formed by PVA modified slurry shows more obvious hydration and cementation characteristics.

**Fig 14:**
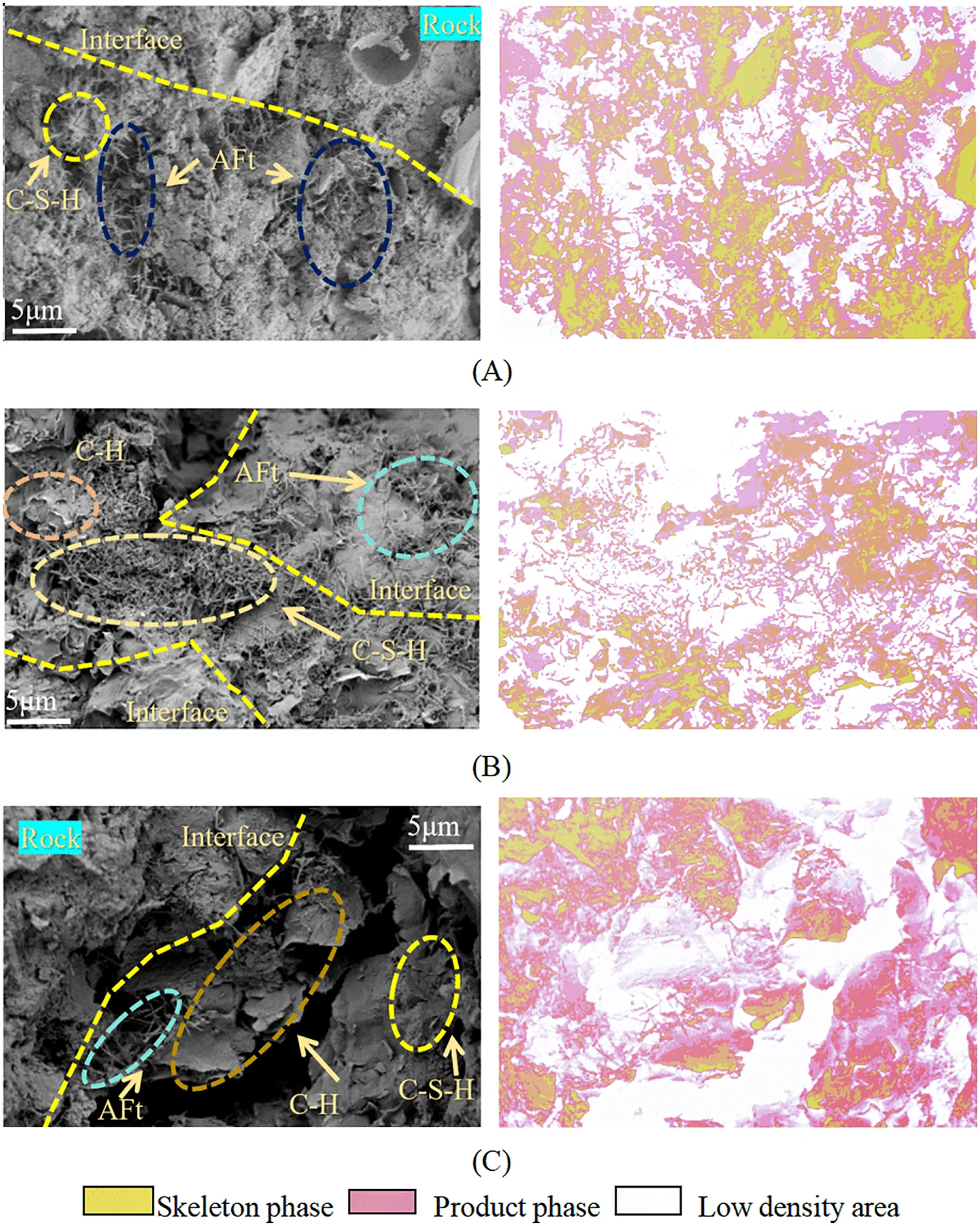
SEM test results of grout-sandstone complex. (a) SEM micrograph and pseudo-colored phase map of sample B1; (b) SEM micrograph and pseudo-colored phase map of sample B2; (c) SEM micrograph and pseudo-colored phase map of sample B3.

After adding PVA, the C-S-H gel, AFt rod crystals and C-H flake crystals on the surface of sandstone and inside the pores and fissures increased significantly, and the encapsulation and filling effect of hydration products on sandstone particles was enhanced. When the PVA content is 0.3%, a certain number of C-S-H gels and AFt crystals appear at the interface, but the spatial distribution is more discrete. The corresponding pseudo-color segmentation results show that the phase continuity of the cementation product is insufficient, and the low-density area accounts for a relatively high proportion, indicating that the low content of PVA has limited effect on interface filling and particle wrapping. When the PVA content is increased to 0.6%, the C-S-H gel at the interface is significantly increased, the phase distribution of the cementation product is more continuous, the encapsulation and filling effect of the hydration product on the surface of the sandstone particles is enhanced, and the interface transition zone is denser. When the PVA content increased to 1.0%, the overall hydration reaction of the system did not stop, and the XRD still showed a higher C-H content and a lower residual C_3_S ratio. However, the SEM results showed that the thicker polymer coating layer, C-H flake crystal enrichment and low density area appeared in the local area, indicating that excessive PVA may reduce the migration efficiency of water and ions at the local interface, making the spatial distribution of hydration products tend to be uneven. At the same time, the proportion of flexible polymer phase increases, which reduces the interface stiffness and effective stress transfer capacity. Therefore, the 1.0% PVA group showed that the overall hydration was still sufficient, but the local interface cementation efficiency and macroscopic bearing capacity were reduced.

As shown in Fig 15, during the hardening process of the grout-sandstone complex, the PVA molecular chain gradually adsorbs, spreads and tangles on the surface of the rock particles and the pore boundary. The local film-like coverage characteristics are observed in the SEM, and it is speculated that PVA may form a local continuous or semi-continuous polymer coating structure on the surface of the particles and the pore boundary.

**Fig 15:**
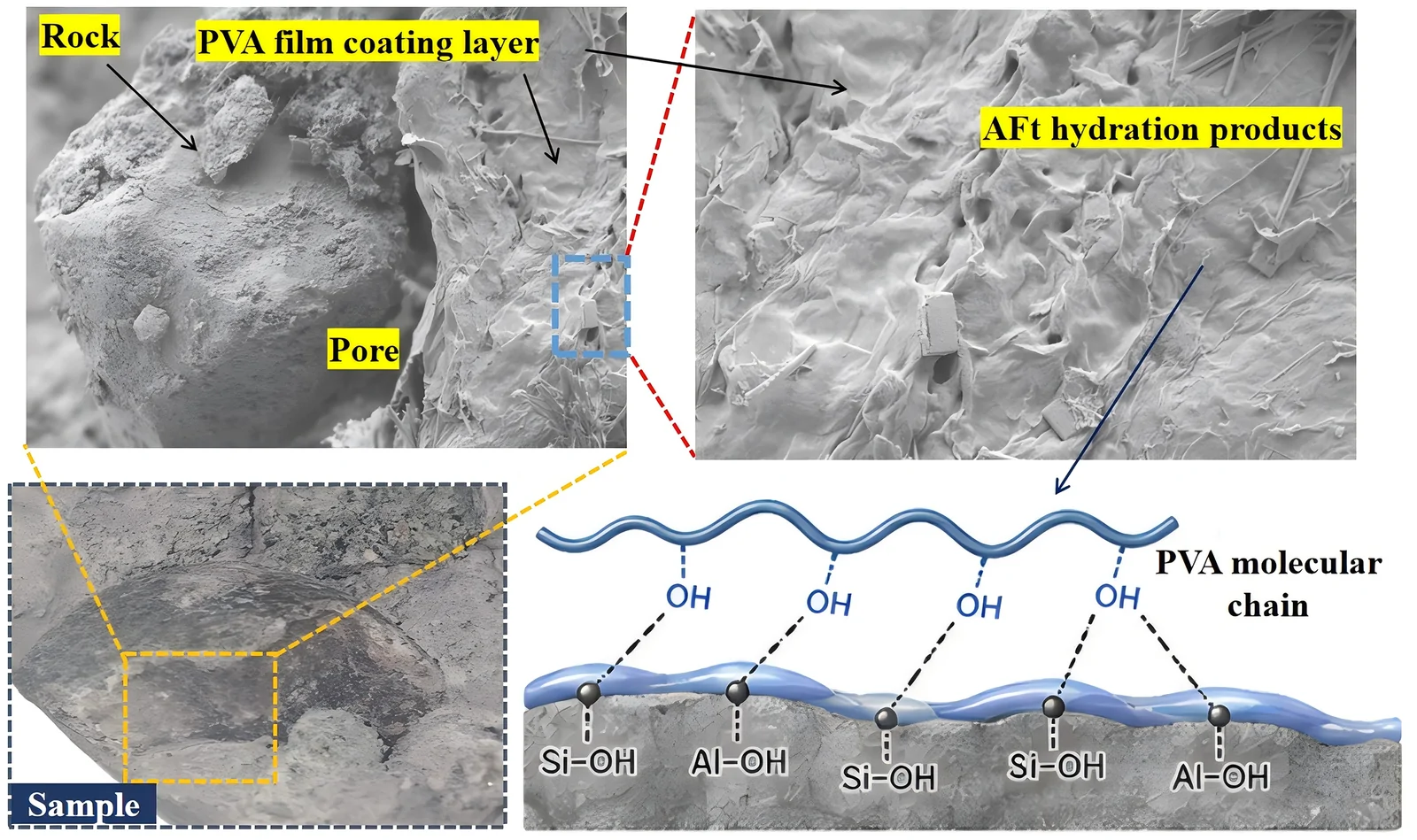
Schematic illustration of the proposed PVA-assisted film-coating mechanism at the grout–sandstone interface.

The PVA flexible membrane enhances the resistance of the slurry to the migration of water molecules. When the PVA content increases to a suitable range, the PVA molecular chain gradually spreads, tangles and forms a continuous or semi-continuous flexible film layer on the pore fissure boundary and the surface of sandstone particles. The film is interwoven with C-S-H gel, C-H crystal and hydration products. On the one hand, it improves the blocking ability of free water migration inside the slurry, and reduces the bleeding segregation and interface water-rich effect. On the other hand, the dispersed rock particles, hydration products and solid waste particles are connected into a continuous composite cementation network, which makes the grouting body change from simple pore filling to a composite bearing structure with interface bonding, fracture sealing and toughness enhancement. The PVA flexible membrane bears the deformation buffering effect, and the hydration products provide rigid skeleton support. The combined effect improves the compactness, bonding stability and sealing property of the grouting composite slurry-rock interface.However, when the content of PVA is excessive, the excessive proportion of polymer phase will reduce the overall stiffness of the composite, and induce the local enrichment of C-H crystals, free water or underreacted particles, forming a relatively loose weak interface area.

## Conclusion

In this paper, through slurry performance test, mechanical test and XRD, FTIR, SEM microscopic characterization, the regulation law of PVA content on the working performance of coal gangue-cement-based grouting material, hydration and cementation of slurry-rock interface and load-bearing enhancement is systematically revealed. The main achievements are as follows:

(1) After the high proportion of coal gangue replaces cement, the fluidity of the slurry is significantly reduced, the setting process is delayed, and the bleeding tendency is enhanced. The fluidity of group A1 and group A2 was 223.00 ± 2.00 mm and 152.67 ± 1.53 mm, respectively. The initial setting time and final setting time of group A2 were 326.67 ± 2.89 min and 420.00 ± 8.66 min, respectively, and the bleeding rate was 2.473 ± 0.015%. After adding PVA, the fluidity of A3-A5 groups increased to 189.67–216.00 mm, and the bleeding rate decreased to 2.027%−1.707%. At the same time, the initial and final setting time was further extended, indicating that PVA could improve the flow stability and water retention performance of high coal gangue slurry, and had a certain delay effect on the setting process.
(2) After the incorporation of PVA, the Ca(OH)_2_, C-S-H related structure and Si-O vibration response in the slurry system were enhanced, and the content of unhydrated C_3_S was reduced. The results of XRD and FTIR showed that an appropriate amount of PVA was beneficial to promote the deposition of hydration products and the development of interfacial cementitious structure. When the PVA content is 0.6%, the Si-O peak shifted toward a higher wavenumber.
(3) The results of uniaxial compression of the slurry-rock composite show that the peak stress of B0, B1, B2 and B3 groups is 4.87 ± 0.14, 5.35 ± 0.06, 6.04 ± 0.09 and 5.12 ± 0.09 MPa, respectively, and the coefficient of variation is 2.82%, 1.14%, 1.45% and 1.76%, respectively. The discreteness between parallel specimens is small. With the increase of PVA content, the peak bearing capacity of the composite increases first and then decreases, and the average peak stress of the 0.6% PVA group is the highest, indicating that PVA has obvious dosage dependence on the mechanical response of the slurry-rock composite. In the test range of this paper, the 0.6% PVA group showed relatively better comprehensive mechanical properties.
(4) SEM observation shows that C-S-H gel, AFt needle rod crystal and C-H flake crystal are more fully distributed on the surface of sandstone particles and near the pore cracks under the condition of appropriate amount of PVA, and the continuity and compactness of the slurry rock interface are improved. When the PVA content is further increased to 1.0%, the local C-H crystal enrichment and the non-uniformity of the interface structure are enhanced, which has an adverse effect on the stiffness and peak bearing capacity of the composite. The modification effect of PVA on the slurry-rock interface is manifested as the synergistic regulation between hydration structure evolution, interface densification and macroscopic mechanical response.
